# A nutritional link to antimicrobial resistance: iron scarcity promotes plasmid co-selection in *Escherichia coli*

**DOI:** 10.1128/aem.01952-25

**Published:** 2026-05-06

**Authors:** Minhyeok Cha, SeonWoo Kim, Jo Ann S. Van Kessel, Bradd J. Haley

**Affiliations:** 1 Environmental Microbial and Food Safety Laboratory, Agricultural Research Service, United States Department of Agriculture 384444 , Beltsville, Maryland, USA; Norwegian University of Life Sciences, Ås, Norway

**Keywords:** iron limitation, siderophore, aerobactin, IncFIB plasmid, antimicrobial resistance, dairy calf gut, isothermal microcalorimetry, plasmid fitness

## Abstract

Multidrug-resistant (MDR) *Escherichia coli* represents a larger fraction of the *E. coli* population in preweaned dairy calf feces compared to older postweaned calves and adult cows. Previous work demonstrated that the MDR genotype is strongly associated with iron-acquisition genes (*iucABCD-iutA* and *sitABCD*), which are primarily carried on IncFIB plasmids that frequently co-encode these systems with antimicrobial-resistance genes in bovine *E. coli*. We hypothesized that iron limitation in the preweaned calf gut favors *E. coli* carrying these iron-acquisition systems, thereby indirectly selecting for antimicrobial resistance. To test this, we quantified growth by isothermal microcalorimetry in bovine cecal medium and used RT-qPCR to measure expression of iron-acquisition genes under iron-replete and iron-limited conditions. Under iron-replete conditions, plasmid carriage imposed a negligible fitness cost. Under iron limitation, IncFIB-positive strains generated up to 3.5-fold more cumulative heat and sustained metabolic activity 6–10 h longer than their plasmid-negative counterparts. This advantage corresponded to strong siderophore gene upregulation, with *iucA* expression increased by up to 38.4-fold. Multivariate analysis further distinguished plasmid-bearing from plasmid-free groups under iron stress. The results of this study demonstrate that plasmid maintenance costs are negligible in rich medium, and they are fully offset by the significant growth advantage conferred by iron-acquisition systems in iron-limited growth conditions. These findings suggest that gut iron availability contributes to the maintenance of antimicrobial resistance in young calves and highlight nutrition-based strategies as potential interventions to reduce antimicrobial resistance in these animals.

**IMPORTANCE:**

The preweaned calf gut is an iron-poor environment that favors *Escherichia coli* carrying IncFIB plasmids encoding both siderophore systems and antimicrobial resistance genes. Our findings show that these plasmids convert a potential liability, the cost of extra DNA, into a fitness advantage under iron limitation. This nutritional-genetic interaction explains why multidrug-resistant strains persist in young calves and suggests that dietary iron management could help reduce antimicrobial resistance in livestock, with implications for food safety and public health.

## INTRODUCTION

Antimicrobial resistance (AMR) is a significant human and animal health issue resulting in an estimated 2.8 million infections and 35,000 deaths and incurs an estimated $4.6 billion in medical treatment costs annually in the United States (1, 2). In human and animal health, the presence of antimicrobial-resistant bacteria is believed to be driven primarily by antibiotic use. In dairy animals, it is well-established that preweaned calves have a higher ratio of antimicrobial-resistant *Escherichia coli* to susceptible *E. coli* than do older animals (3, 4). This occurs even in the absence of antibiotic administration, indicating that the age disparity in antimicrobial-resistant *E. coli* carriage is not driven by antimicrobial use in these calves. Previous work demonstrated that the multidrug-resistant (MDR) genotype in randomly selected *E. coli* from dairy animals is strongly associated with the *iucABCD-iutA* (aerobactin) and *sitABCD* (*Salmonella* iron transporter) operons, suggesting a link between iron availability and the selection of antimicrobial resistance (5). However, genomic analyses only establish a correlation and do not directly prove a causal link between this genetic association and the fitness of bacteria in iron-deficient environments (6–8).

Iron is essential for cellular processes such as electron transport and DNA synthesis. To meet this requirement, *E. coli* encodes an extensive network of encoded siderophore receptors and iron uptake pathways (9). But bioavailable iron is scarce in the mammalian gut due to sequestration by host proteins (10). This limitation is particularly pronounced in preweaned calves, whose diet of colostrum and milk is iron-poor compared to the more iron-rich diets of grains and forages of older animals. Under such iron-limited conditions, bacteria with efficient siderophore-mediated iron acquisition are expected to outcompete bacteria lacking these accessory genes (11).

IncFIB plasmids are frequently carried by members of the Enterobacteriaceae, where they are known to encode metal, biocide, and antimicrobial resistance genes (12–15). In addition to these functions, these plasmids frequently encode plasmid-borne siderophore systems, such as *sitABCD* and *iucABCD-iutA*, which can enhance iron acquisition under iron-limited conditions (5). This co-localization of AMR and iron-acquisition genes provides a potential selective advantage for resistant bacteria in low-iron environments like the preweaned dairy calf gut, thus representing a potential target for mitigating calf gut colonization by antimicrobial-resistant strains (16–20).

This study was designed to describe the contribution of IncFIB-encoded iron-acquisition systems to the competitive fitness of *E. coli* under iron-deficient conditions. The objective was to investigate mechanistic relationships between the ecological pressure of iron limitation and the persistence of MDR bacterial populations, potentially providing the basis for developing targeted interventions.

## RESULTS

### Expression analysis of siderophore genes (*sitA, iutA,* and *iucA*)

**Fig 1:**
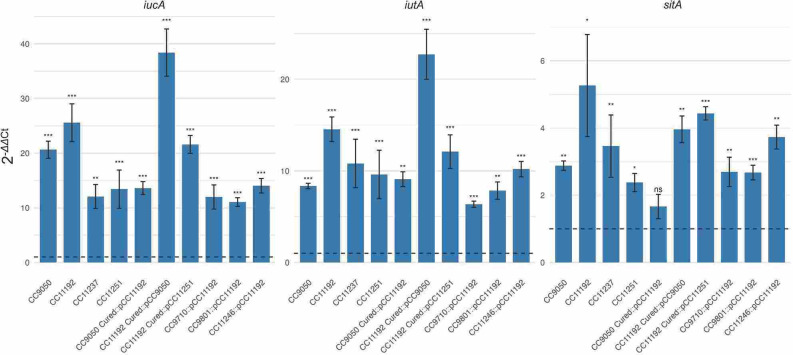
Iron limitation sharply upregulates plasmid-encoded siderophore genes. The relative transcript abundance of *iucA****,*** *iutA,* and *sitA* (left → right panels) in 10 representative strains. Cultures were grown for 4 h at 37 °C either in normal cecal medium or in the same medium rendered iron-depleted with 400 µM 2,2′-dipyridyl. Transcript levels were quantified by qPCR, normalized to the stable housekeeping reference *gyrA*, and expressed as relative transcript abundance 2^-^*^ΔΔ^*^Ct^. Each bar is the arithmetic mean of triplicates; error bars represent the propagated standard error of the mean (√ΣSE^2^). Strains are ordered left-to-right as follows: CC9050, CC11192, CC11237, CC11251, CC9050-Cured::pCC11192, CC11192-Cured::pCC9050, CC11192-Cured::pCC11251, CC9710::pCC11192, CC9801::pCC11192, and CC11246::pCC11192. Error bars denote propagated standard errors. A dashed horizontal line at 1 indicates no change. Asterisks mark significant differences between media for a given strain determined by Welch’s *t*-test on replicate *Δ*Ct values (*P* < 0.05; *, P < 0.01; **, P < 0.001; ns, not significant).

When analyzed via qPCR, transcript levels of *sitA*, *iutA*, and *iucA* in the wild-type strains were significantly upregulated under iron-depleted conditions compared to iron-replete conditions (Fig. 1). Similar differences were consistently observed for each transconjugant. The greatest difference in expression was observed for *iucA*, which was 11.0-fold to 38.4-fold higher across all strains. Expression of *iutA* and *sitA* was 6.3-fold to 22.7-fold higher and 1.6-fold to 5.3-fold higher, respectively, in the iron-depleted conditions vs. the iron-replete conditions. These observed differences were generally significant (*P* < 0.01, two-tailed Welch’s *t*-test; Fig. 1). A similar trend of increased *sitA* expression was observed in strain CC9050Cured::pCC11192 under iron-depleted conditions; however, this difference did not reach statistical significance (*P* > 0.05) due to high inter-replicate variance.

### IMC profiles of wild-type strains in response to iron availability

**Fig 2:**
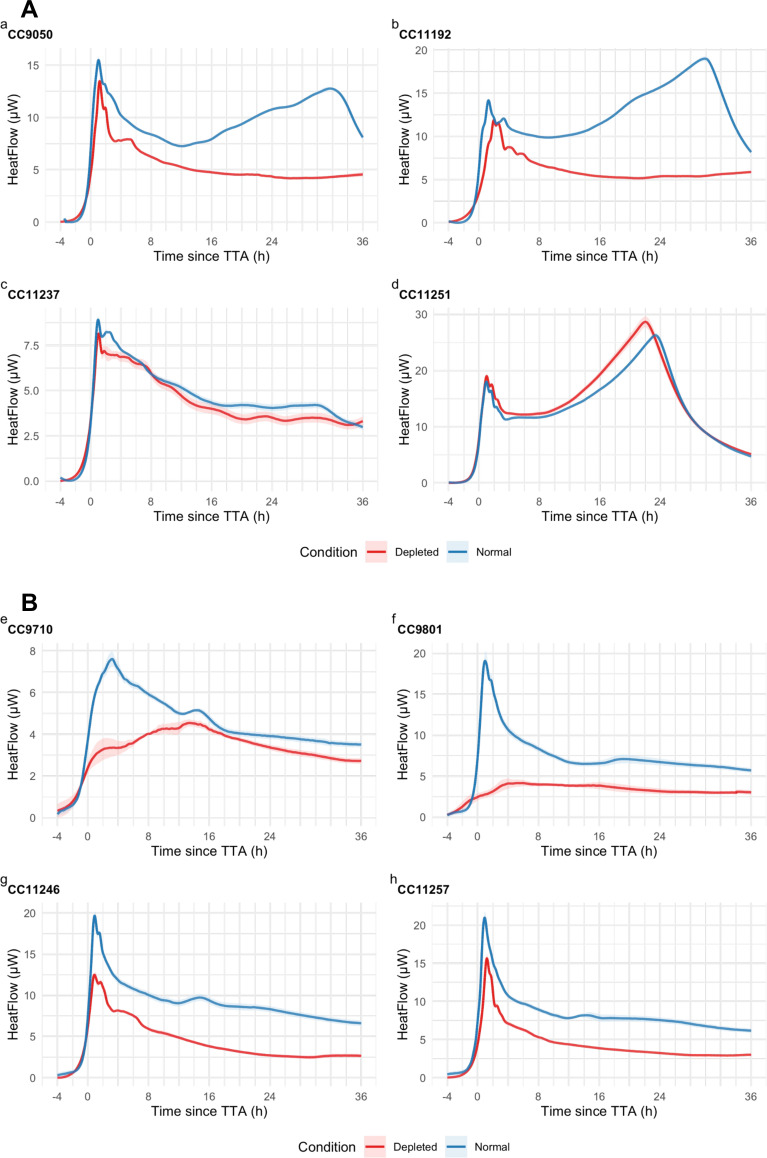
Wild-type MDR *E. coli* harboring IncFIB/iron-acquisition plasmids are more resilient to iron limitation. Real-time heat-flow profiles of representative *E. coli* strains grown in normal (blue) or iron-depleted (red) cecal medium. Curves represent the mean ± SEM of three biological replicates. (**A**) MDR wild-type strains carrying the IncFIB plasmid (CC9050, CC11192, CC11237, CC11251). CC9050 and CC11192: the primary heat-flow peak falls from ~16 µW and ~14 µW (Normal) to ~13 µW and ~11 µW (Depleted), respectively. (**B**) Sensitive wild-type strains lacking IncFIB plasmids (CC9710, CC9801, CC11246, and CC11257). Across all plasmid-negative strains, the heat flow remains consistently lower in the iron-depleted medium than in the normal medium throughout the measurement period, with no recovery of peak intensity at later time points.

When the MDR plasmid-positive wild-type (WT) strains were examined for resilience to iron limitation, the heat production profiles for CC11237 and CC11251 were very similar when grown in either iron-replete or iron-depleted medium, and the primary peaks were similar (8.9 µW vs. 8.3 µW and 18.8 µW vs. 18.1 µW, respectively) (Fig. 2A). While the peak heat flow for the other two MDR strains, CC9050 and CC11192, was lower in iron-depleted than in iron-replete media (13.5 µW vs. 15.5 µW and 10.2 µW vs. 14.2 µW, respectively), their overall metabolic activity was maintained to a greater degree than that of the plasmid-negative strains described below.

In contrast, heat production by the plasmid-negative, antimicrobial-susceptible strains was consistently lower in iron-depleted than in iron-replete medium (Fig. 2B). All four strains produced less heat in iron-depleted than in iron-replete media. For strains CC9710 and CC9801, the primary peak was not well resolved. While CC11246 and CC11257 generated well-defined primary peaks under depletion, their peak amplitudes were lower in iron-depleted than in iron-replete medium (12.0 vs. 20.2 µW and 15.7 vs. 21.2 µW, respectively), and the post-peak heat flow remained consistently lower thereafter.

A key difference among the plasmid-positive strains was observed in the late-growth secondary heat production peak. This peak, observed when CC9050, CC11192, and CC11251 were grown in normal media, was absent under iron depletion for CC9050 and CC11192. Uniquely, the secondary heat production peak in CC11251 not only persisted but was also intensified and appeared earlier under iron-depleted conditions, indicating a strain-specific enhancement of late-phase metabolic activity (Fig. 2A).

### Growth kinetics by plasmid status under iron-limited conditions

**Fig 3:**
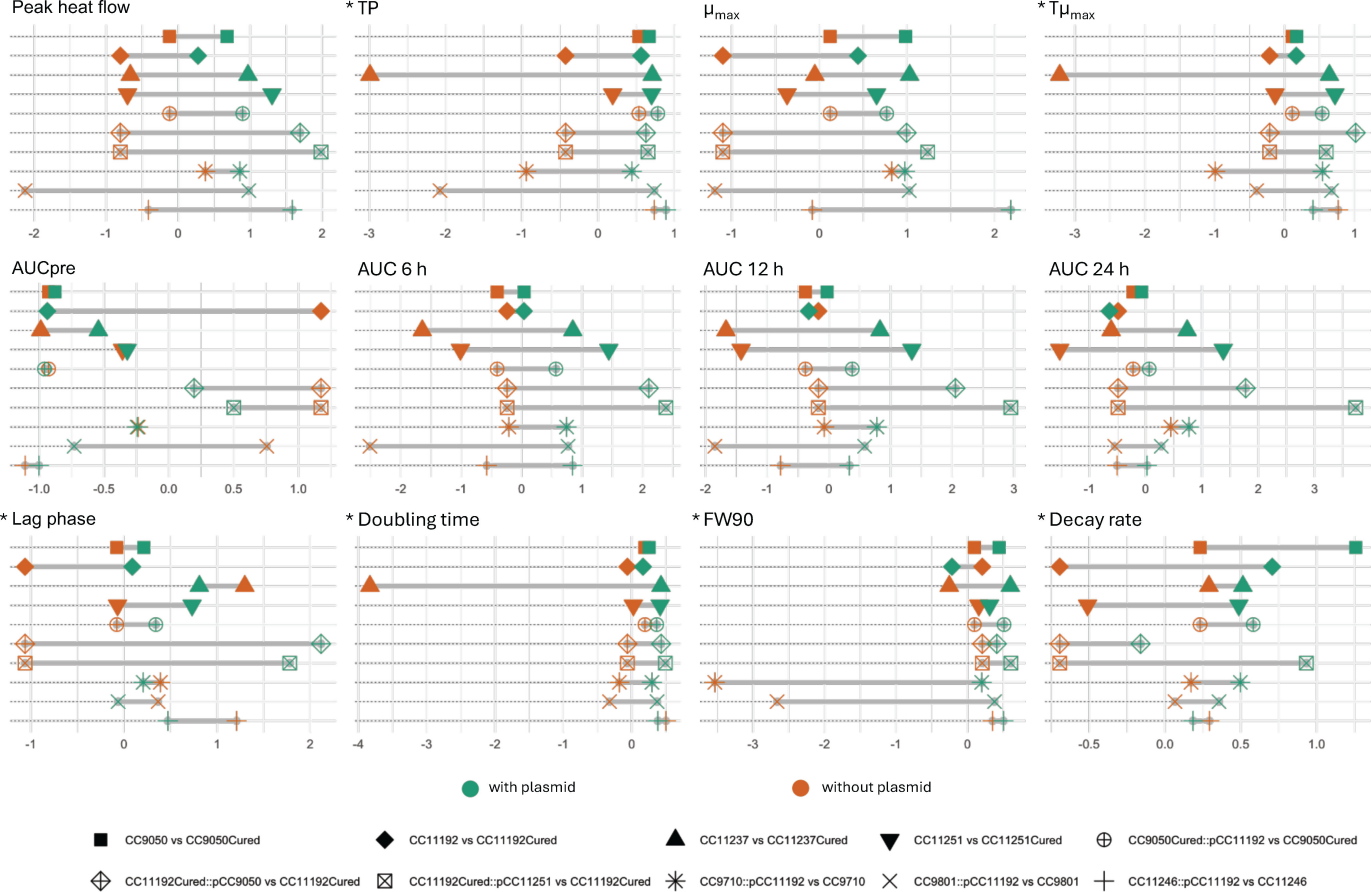
Quantitative analysis of isogenic pairs confirm that IncFIB plasmids carrying iron-acquisition systems confer a direct fitness advantage. Dumbbell plots comparing 12 Z-score–standardized growth kinetic metrics between 10 isogenic plasmid-positive (green) and plasmid-negative (orange) strain pairs. Each point shape represents a unique strain pair. The value for each metric represents the response to iron stress, calculated as the difference between iron-depleted and normal conditions (*Δ* = Depleted – Normal). Full numerical data are provided in Table 1. The 12 metric panels are arranged left-to-right, top-to-bottom as follows: (i) peak heat flow, (ii) TP, (iii) µ_max_, (iv) Tµ_max_, (v) AUCpre, (vi) AUC 6 h, (vii) AUC 12 h, (viii) AUC 24 h, (ix) lag phase, (v) doubling time, (xi) FW90, and (xii) decay rate. In each panel, a rightward shift of the green point relative to the orange point indicates a positive plasmid effect (i.e., enhanced performance under iron stress). Metrics marked with an asterisk (*) were direction-aligned by inverting those for which lower raw values indicate better growth performance (e.g., lag phase, doubling time), so that higher values and rightward shifts consistently represent a relative fitness advantage of plasmid-bearing strains under iron stress. Abbreviations: TTA, time to activate; TP, time to peak heat flow relative to TTA; μ_max_, maximum rate of increase in heat flow; Tμ_max_, time of μ_max_; AUC, Area Under the Curve; AUCpre, pre-peak area under the curve; and FW90, Full width at 90% of peak height.

Under iron-limited conditions, the heat-flow profiles of isogenic plasmid-bearing and plasmid-cured strains differed significantly according to plasmid status. To quantify the metabolic differences observed in the heat-flow profiles, 12 growth-kinetic metrics were compared between 10 isogenic strain pairs (Table 1, Fig. 3). This analysis revealed that plasmid carriage consistently and significantly enhanced the majority of these metrics across the strain pairs tested.

**TABLE 1:** Differential growth metrics (*Δ* = Depleted – Normal) for plasmid-positive and plasmid-negative strain pairs^*a*^^,^^*b*^^,^^*c*^

	Peak heat flow (μW)			TP (s)			μmax (s^−1^)			Tμmax (s)		
Pair	+	−		+	−		+	−		+	−	
CC9050 vs. CC9050Cured	−2.02 ± 0.73	−5.84 ± 0.68	***	380.27 ± 260.48	1,322.66 ± 989.83	**	−1.85×10^−3^ ± 3.00×10^−4^	−2.20×10^−4^ ± 4.12×10^−4^	***	2,776.27 ± 151.35	3,097.32 ± 879.76	ns
CC11192 vs. CC11192Cured	−3.95 ± 1.57	−9.10 ± 1.51	***	1,121.21 ± 950.45	8,069.96 ± 817.03	***	−4.18×10^−3^ ± 8.67×10^−4^	−1.25×10^−3^ ± 2.13×10^−4^	***	2,803.87 ± 403.55	4,709.29 ± 1,538.94	***
CC11237 vs. CC11237Cured	−0.63 ± 0.37	−8.44 ± 0.36	***	96.14 ± 452.45	26,058.78 ± 11,030.57	***	−2.19×10^−3^ ± 5.91×10^−5^	−1.33×10^−4^ ± 2.41×10^−4^	***	435.47 ± 426.85	19,725.11 ± 9,519.96	***
CC11251 vs. CC11251Cured	0.96 ± 0.74	−8.63 ± 0.25	***	159.03 ± 139.21	3,736.35 ± 1,223.49	***	−2.79×10^−3^ ± 2.42×10^−4^	−8.49×10^−4^ ± 1.87×10^−4^	***	36.70 ± 55.00	4,321.02 ± 564.30	***
CC9050Cured::pCC11192 vs. CC9050Cured	−0.99 ± 0.34	−5.84 ± 0.68	***	−416.71 ± 180.13	1,322.66 ± 989.83	***	−1.85×10^−3^ ± 3.00×10^−4^	−6.29×10^−4^ ± 7.65×10^−5^	***	950.62 ± 399.95	3,097.32 ± 879.76	***
CC11192Cured::pCC9050 vs. CC11192Cured	2.82 ± 0.73	−9.10 ± 1.51	***	679.48 ± 989.71	8,069.96 ± 817.03	***	−4.18×10^−3^ ± 8.67×10^−4^	−1.99×10^−4^ ± 3.18×10^−4^	***	−1,443.85 ± 997.99	4,709.29 ± 1,538.94	***
CC11192Cured::pCC11251 vs. CC11192Cured	4.21 ± 0.84	−9.10 ± 1.51	***	487.79 ± 1,883.04	8,069.96 ± 817.03	***	−4.18×10^−3^ ± 8.67×10^−4^	2.57×10^−4^ ± 3.92×10^−4^	***	664.12 ± 1,647.70	4,709.29 ± 1,538.94	***
CC9710::pCC11192 vs. CC9710	−1.17 ± 0.55	−3.46 ± 0.68	***	1,974.96 ± 2,406.54	11,672.44 ± 9,922.19	*	−5.22×10^−4^ ± 1.98×10^−4^	−2.35×10^−4^ ± 1.61×10^−4^	**	922.29 ± 365.33	8,602.44 ± 13,430.28	ns
CC9801::pCC11192 vs. CC9801	−0.58 ± 0.83	−15.43 ± 0.99	***	−84.13 ± 188.12	19,610.88 ± 8,324.16	***	−4.35×10^−3^ ± 1.67×10^−4^	−1.42×10^−4^ ± 1.76×10^−4^	***	281.20 ± 240.01	5,634.88 ± 5,231.08	*
CC11246::pCC11192 vs. CC11246	2.31 ± 0.49	−7.24 ± 0.76	***	−1,164.26 ± 232.48	−99.15 ± 675.85	***	−2.24×10^−3^ ± 1.89×10^−4^	2.06×10^−3^ ± 2.91×10^−4^	***	1,609.40 ± 282.53	−191.49 ± 712.92	***

	AUCpre (μJ)			AUC (0–6 h) (μJ)			AUC (0–12 h) (μJ)			AUC (0–24 h) (μJ)		
	+	-		+	-		+	-		+	-	
CC9050 vs. CC9050Cured	−9,328.05 ± 2,136.55	−10,493.90 ± 4,359.98	ns	−57,917.56 ± 3,355.18	−82,643.95 ± 7,731.22	***	−102,220.34 ± 6,921.18	−137,553.12 ± 13,788.37	***	−279,984.73 ± 12,445.76	−325,069.26 ± 32,375.12	***
CC11192 vs. CC11192Cured	−10,725.05 ± 12,604.05	39,846.61 ± 4,600.95	***	−58,122.08 ± 5,586.21	−73,574.06 ± 15,581.22	***	−132,029.17 ± 11,039.51	−116,383.93 ± 33,211.88	*	−450,007.90 ± 22,440.69	−404,554.60 ± 109,989.50	ns
CC11237 vs. CC11237Cured	−1,290.89 ± 1,879.18	−11,941.78 ± 4,421.68	***	−13,239.58 ± 9,761.72	−151,336.98 ± 7,885.62	***	−16,364.64 ± 19,687.42	−266,286.19 ± 15,127.86	***	−39,083.30 ± 40,766.57	−440,981.53 ± 19,545.03	***
CC11251 vs. CC11251Cured	4,040.65 ± 3,980.61	3,163.98 ± 11,208.22	ns	19,765.07 ± 10,404.29	−116,353.94 ± 2,349.87	***	35,342.27 ± 23,407.55	−241,760.79 ± 4,791.73	***	151,682.66 ± 71,066.79	−713,015.67 ± 17,708.06	***
CC9050Cured::pCC11192 vs. CC9050Cured	−11,260.38 ± 1,844.03	−10,493.90 ± 4,359.98	ns	−28,783.66 ± 7,383.71	−82,643.95 ± 7,731.22	***	−61,308.80 ± 13,981.63	−137,553.12 ± 13,788.37	***	−240,135.38 ± 27,922.29	−325,069.26 ± 32,375.12	***
CC11192Cured::pCC9050 vs. CC11192Cured	16,379.39 ± 7,665.91	39,846.61 ± 4,600.95	***	56,431.29 ± 8,470.55	−73,574.06 ± 15,581.22	***	106,118.03 ± 15,939.00	−116,383.93 ± 33,211.88	***	270,441.80 ± 36,031.30	−404,554.60 ± 109,989.50	***
CC11192Cured::pCC11251 vs. CC11192Cured	23,726.99 ± 11,815.06	39,846.61 ± 4,600.95	**	72,196.35 ± 9,503.98	−73,574.06 ± 15,581.22	***	195,929.60 ± 19,939.42	−116,383.93 ± 33,211.88	***	851,667.56 ± 41,419.81	−404,554.60 ± 109,989.50	***
CC9710::pCC11192 vs. CC9710	5,872.42 ± 18,036.92	6,078.70 ± 33,628.31	ns	−18,990.35 ± 6,436.05	−71,899.75 ± 14,204.01	***	−21,453.81 ± 13,786.63	−106,886.11 ± 14,389.70	***	−29,905.08 ± 27,530.11	−125,743.13 ± 23,877.89	***
CC9801::pCC11192 vs. CC9801	−5,773.80 ± 529.55	29,823.17 ± 19,503.65	***	−17,578.78 ± 5,964.22	−199,338.54 ± 20,120.63	***	−41,314.98 ± 5,519.89	−284,357.51 ± 31,218.73	***	−176,178.46 ± 21,390.84	−421,922.22 ± 60,253.06	***
CC11246::pCC11192 vs. CC11246	−12,267.27 ± 935.36	−14,844.46 ± 6,141.75	ns	−13,455.89 ± 11,332.46	−92,137.43 ± 9,646.58	***	−66,030.90 ± 31,390.51	−177,560.61 ± 23,735.40	***	−251,664.35 ± 65,126.77	−409,459.54 ± 51,590.25	**

	Lag phase (s)			Doubling time (s)			FW90 (s)			Decay Rate (s^−1^)		
	+	-		+	-		+	-		+	-	
CC9050 vs. CC9050Cured	7,784.73 ± 1,741.94	10,640.68 ± 4,937.32	*	1,320.60 ± 338.11	1,760.32 ± 1,370.87	ns	−853.00 ± 614.04	3,385.00 ± 1,205.87	***	1.04×10^−4^ ± 2.90×10^−6^	−2.51×10^−4^ ± 6.96×10^−5^	***
CC11192 vs. CC11192Cured	9,005.46 ± 2,552.95	20,325.04 ± 4,135.56	***	1,960.54 ± 638.19	3,600.63 ± 592.01	***	7,252.67 ± 4,779.33	2,039.67 ± 198.30	*	4.23×10^−4^ ± 4.65×10^−4^	−6.07×10^−5^ ± 7.01×10^−5^	***
CC11237 vs. CC11237Cured	1,926.86 ± 2,817.76	−2,868.11 ± 2,866.21	*	70.47 ± 534.74	30,657.45 ± 24,161.12	**	−2,788.00 ± 3,407.79	7,705.67 ± 2,200.47	***	8.35×10^−5^ ± 5.14×10^−6^	6.14×10^−6^ ± 1.83×10^−5^	***
CC11251 vs. CC11251Cured	2,694.30 ± 1,350.44	10,559.98 ± 5,030.10	**	158.37 ± 37.50	2,977.35 ± 538.35	***	769.67 ± 748.90	2,630.33 ± 2,050.00	*	3.60×10^−4^ ± 2.23×10^−4^	1.53×10^−5^ ± 2.05×10^−4^	**
CC9050Cured::pCC11192 vs. CC9050Cured	6,522.05 ± 1,485.62	10,640.68 ± 4,937.32	**	521.95 ± 40.61	1,760.32 ± 1,370.87	**	−1,714.00 ± 1,828.46	3,385.00 ± 1,205.87	***	1.04×10^−4^ ± 2.90×10^−6^	−1.75×10^−5^ ± 8.70×10^−5^	**
CC11192Cured::pCC9050 vs. CC11192Cured	−10,918.48 ± 5,925.14	20,325.04 ± 4,135.56	***	57.48 ± 896.03	3,600.63 ± 592.01	***	−469.00 ± 1,611.87	2,039.67 ± 198.30	**	4.23×10^−4^ ± 4.65×10^−4^	2.39×10^−4^ ± 2.05×10^−4^	ns
CC11192Cured::pCC11251 vs. CC11192Cured	−7,608.12 ± 2,747.27	20,325.04 ± 4,135.56	***	−389.88 ± 1,766.57	3,600.63 ± 592.01	***	−2,879.67 ± 487.45	2,039.67 ± 198.30	***	4.23×10^−4^ ± 4.65×10^−4^	−1.39×10^−4^ ± 5.42×10^−5^	***
CC9710::pCC11192 vs. CC9710	7,848.37 ± 706.83	6,043.89 ± 10,574.72	ns	1,016.96 ± 798.32	4,447.77 ± 6,788.05	ns	2,120.00 ± 2,882.31	48,038.67 ± 18,003.89	***	1.24×10^−4^ ± 6.04×10^−6^	1.19×10^−5^ ± 2.67×10^−5^	***
CC9801::pCC11192 vs. CC9801	10,506.80 ± 1,883.35	6,282.12 ± 5,586.53	ns	478.53 ± 75.56	5,477.88 ± 2,428.52	***	−71.33 ± 1,600.87	37,326.67 ± 24,334.98	**	1.61×10^−4^ ± 2.50×10^−5^	6.06×10^−5^ ± 8.82×10^−5^	**
CC11246::pCC11192 vs. CC11246	5,246.26 ± 2,905.52	−2,006.85 ± 4,288.35	**	395.74 ± 213.39	−451.49 ± 779.56	**	−1,609.67 ± 461.68	261.00 ± 59.85	***	8.24×10^−5^ ± 5.80×10^−5^	1.20×10^−4^ ± 3.53×10^−5^	**

^*a*^ All values represent the change (*Δ*) in each metric, calculated as the value in the iron-depleted medium minus the value in the normal medium (*Δ* = Depleted – Normal). Data are shown as the mean ± standard error of the mean (SEM) from three biological replicates. The “+” column indicates the plasmid-bearing strain, and the “–” column indicates its corresponding plasmid-negative counterpart. Statistical significance of the difference between the “+” and “–” strains for each metric was determined by a two-tailed Welch’s *t*-test.

^*b*^ TTA, time to activate; TP, time to peak heat flow relative to TTA; μ_max_, maximum rate of increase in heat flow; Tμ_max_, time of μ_max_; AUC, Area Under the Curve; AUCpre, pre-peak area under the curve; FW90, Full width at 90% of peak height.

^*c*^ *P* < 0.05; *P < 0.01; **P < 0.001; ns, not significant.

Among the 12 metrics examined, significant differences were observed in all 10 strain pairs (*P* < 0.05) for peak heat flow, relative peak time, *μ_max_*, the area under the heat-flow curve (*AUC_0-6h_* and *AUC_0-12h_*), and the duration the signal remained above 90% of the maximum (FW90) (Fig. 3). For each of these parameters, plasmid-bearing strains exhibited higher values than their plasmid-negative counterparts. Significant differences were observed for other metrics, including doubling time and decay rate in most, but not all, pairs. Detailed numerical results and overall trends are provided in Table 1, and the Z-score dumbbell plots are in Fig. 3, respectively. Raw heat-flow profiles for each isogenic strain pair are available in Fig. S2.

### Effect of plasmid carriage on early-phase growth patterns under iron-limited conditions: integrated PCA and qPCR analysis

**Fig 4:**
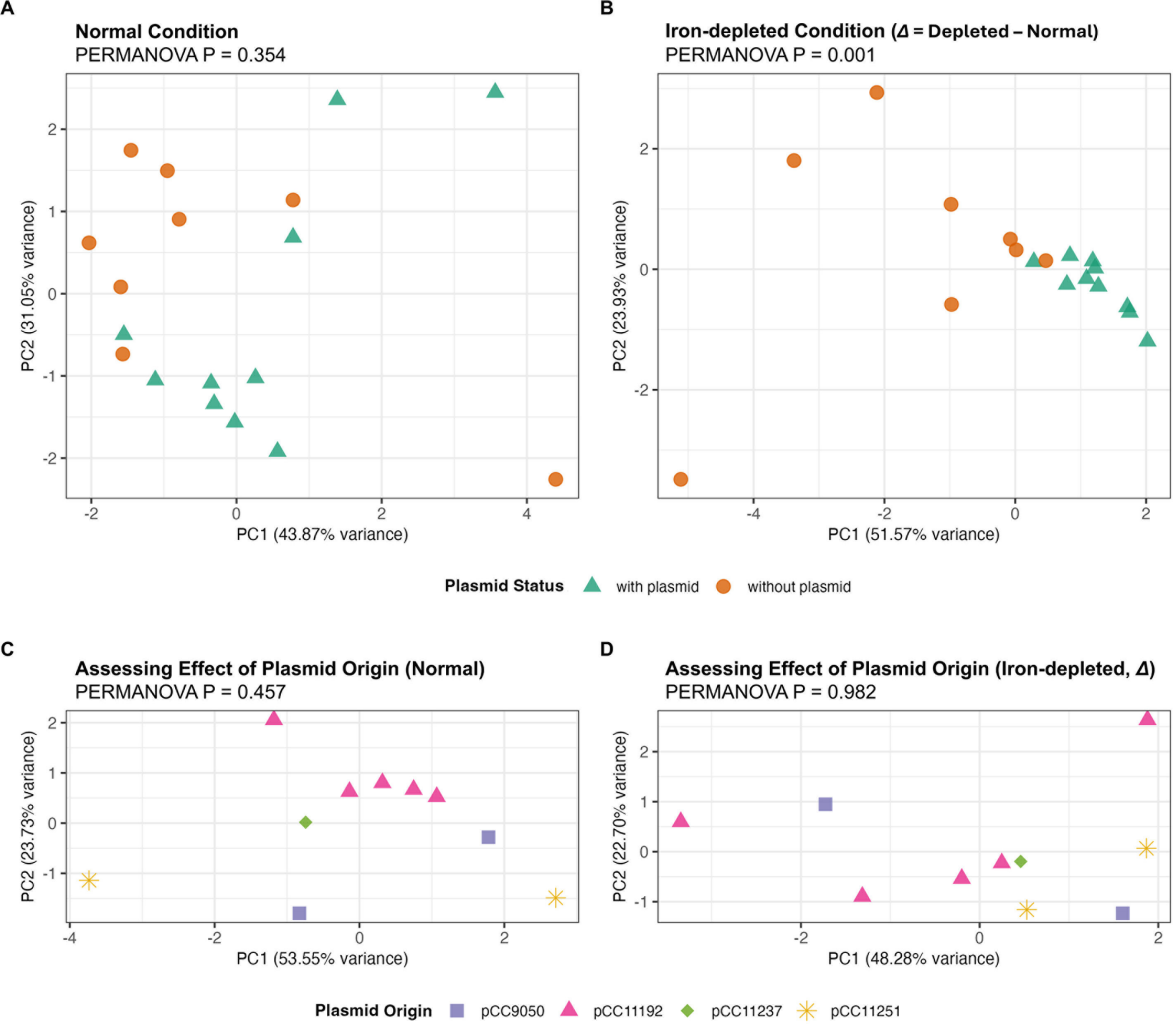
Plasmid carriage is the dominant factor separating metabolic profiles under iron limitation. PCA of key early-growth metrics derived from the mean of three biological replicates. (**A**) Under normal (iron-replete) conditions, strains with (turquoise triangles) and without (orange circles) plasmids form an overlapping cluster, showing no significant separation (PERMANOVA *P* = 0.354). (**B**) In contrast, analysis of the metabolic response to iron stress (*Δ* = Depleted – Normal) shows a clear and significant separation of the two groups primarily along the PC1 axis (PERMANOVA *P* = 0.001). (**C and D**) A subsequent PCA performed only on plasmid-positive strains, integrating growth metrics with *iucA* gene expression, reveals no distinct sub-clustering based on plasmid origin in either normal (**C**) or iron-limited (**D**) conditions. The legend indicates the strain origin of the harbored plasmid.

Principal component analysis (PCA) was performed on seven descriptors of early growth: relative time of peak heat flow (TP), peak heat flow, lag phase, *μ_max_*, relative time of μ_max_ (*Tμ_max_*), doubling time, and pre-peak area under the curve (*AUC_pre_*), after Z-score scaling. Three biological replicates from the same chromosomal background were averaged so that each strain was represented by a single point. Comprehensive PCA loadings and PERMANOVA *P*-values for Fig. 4 data sets were described in Table 2.

**TABLE 2:** Comprehensive PCA loadings and PERMANOVA *P*-values for Figure 4 data sets^*a*^^,^^*b*^

Data set	Variable	PC1 loading	PC2 loading	Total contribution	PERMANOVA *P*
All strains					
Normal, heat-flow only	TP	−0.44	0.36	0.31	0.354
	Tμmax	−0.26	−0.58	0.29	
	μmax	−0.45	0.25	0.28	
	Doubling time	−0.49	−0.18	0.27	
	Lag phase	−0.28	−0.47	0.27	
	Peak heat flow	−0.46	0.18	0.26	
	AUCpre	0.03	−0.42	0.14	
Depleted – Normal, heat-flow only	Doubling time	−0.43	0.42	0.32	0.001
	Tμmax	−0.46	0.33	0.31	
	Peak heat flow	−0.44	−0.32	0.30	
	μmax	−0.38	−0.43	0.30	
	TP	−0.49	0.15	0.29	
	Lag phase	−0.11	−0.48	0.17	
	AUCpre	0.12	0.43	0.16	
Plasmid-positive strains only					
Normal with heat-flow + qPCR	Tμmax	0.44	0.49	0.44	0.457
	TP	0.30	−0.54	0.38	
	μmax	0.50	−0.34	0.36	
	Doubling time	0.45	0.34	0.32	
	Peak heat flow	0.47	−0.27	0.29	
	iucA	−0.20	−0.41	0.21	
Depleted – Normal with heat-flow + qPCR	Tμmax	0.40	−0.57	0.48	0.982
	TP	0.37	0.59	0.48	
	μmax	0.55	−0.22	0.35	
	Doubling time	0.55	−0.02	0.30	
	Peak heat flow	0.32	0.44	0.29	
	iucA	−0.02	−0.31	0.09	

^*a*^ Values indicate the contribution of each metric to PC1 and PC2 for the four PCA analyses shown in Fig. 4. "Total contribution" represents the combined squared loadings on PC1 and PC2, indicating the overall importance of each variable in the two-dimensional space.

^*b*^ TTA, time to activate; TP, time to peak heat flow relative to TTA; μ_max_, maximum rate of increase in heat flow; T_μmax_, time of μ_max_; AUC, Area Under the Curve; AUCpre, pre-peak area under the curve; and FW90, Full width at 90% of peak height. PERMANOVA *P*-values are based on Euclidean distances computed from the same scaled data.

Under iron-replete conditions, PC1 and PC2 explained 43.87% and 31.05% of the variance, respectively (cumulative 74.92%). The five largest contributors were TP (–0.44/0.36), *Tμ_ma_*_x_ (–0.26/–0.58), *μ_max_* (–0.45/0.25), doubling time (–0.49/–0.18), and lag phase (–0.28/–0.47). Plasmid-positive and plasmid-negative strains overlapped on the PC1 versus PC2 plane, and no statistical difference was detected, indicating no separation between plasmid-positive strains and plasmid-negative strains under normal growth conditions (PERMANOVA *P* = 0.354) (Fig. 4A). This finding suggests that under ideal, iron-replete conditions, the presence of the plasmid does not impose a significant metabolic burden on the host.

In contrast to normal conditions, when iron was limited (Fig. 4B, using the *Δ* values defined as Depleted minus Normal), there was a highly significant separation in the combined growth metrics between plasmid-positive and plasmid-negative strains. This differential approach allows for the isolation of the specific bacterial response to the stressor by effectively removing background growth noise, which explains the heightened separation observed compared to iron-replete conditions. PC1 and PC2 accounted for 51.57% and 23.93% of the variance (cumulative 75.50%). The variables with the largest absolute loadings were doubling time (–0.43/+0.42), Tμ_max_ (–0.46/+0.33), peak heat flow (–0.44/–0.32), μ_max_ (–0.38/–0.43), and TP (–0.49/+0.15). These loadings indicate that strains with a negative PC1 score, such as the plasmid-cured strains, experienced impaired growth kinetics, characterized by significantly prolonged doubling times and reduced peak heat flow in response to iron depletion. This result indicates that plasmid carriage confers a metabolic advantage to the host under iron-limited conditions. Plasmid-positive and plasmid-negative strains were clearly separated along PC1, with a significant group effect (PERMANOVA *P* = 0.001) (Fig. 4B).

For plasmid-positive strains, the five top-loading variables were combined with the siderophore gene *iucA* (highest log-fold change) and re-analyzed. In the Normal data set, PC1 and PC2 explained 53.55% and 23.73% of the variance (total 77.28%). PC1 was driven by μ_max_ (+0.50), peak heat flow (+0.47), doubling time (+0.45), Tμ_max_ (+0.44), and TP (+0.30), and PC2 was dominated by TP (–0.54) and Tμ_max_ (+0.49). The loading of relative expression of *iucA* (–0.21/–0.41) did not place it among the five highest-loading variables, and plasmid sub-types did not form separate clusters (PERMANOVA *P* = 0.457) (Fig. 4C). This lack of separation suggests that under iron-replete conditions, plasmid sub-type and the high expression of the *iucA* gene are not the primary drivers of metabolic differences between these strains.

Applying the same variable set to the *Δ* matrix yielded PC1 and PC2 variances of 48.28% and 22.70% (cumulative 70.98%). PC1 received coherent positive contributions from μ_max_ (+0.55), doubling time (+0.55), Tμ_max_ (+0.40), TP (+0.37), and peak heat flow (+0.32). PC2 contrasted TP (+0.59) with Tμ_max_ (–0.57). The *iucA* loading (–0.02/–0.31) lay outside the top five, and no statistically significant group separation was observed (PERMANOVA *P* = 0.982) (Fig. 4D). This finding indicates that even under iron-limited conditions, the specific plasmid subtype does not explain the differences in how the strains respond, and instead, the overall presence of a plasmid is the key factor.

In summary, the multivariate analysis confirmed that plasmid carriage was the dominant factor separating the metabolic profiles under iron limitation, although no distinct sub-clusters were observed among the plasmid-positive strains.

## DISCUSSION

This study combined high-resolution isothermal microcalorimetry with gene expression analysis to elucidate the functional impact of IncFIB plasmids on *E. coli* growth under iron-limited conditions. We focused on the aerobactin biosynthesis operon (*iucABCD-iutA*) and the *Salmonella* iron transport system (*sitABCD*), which are frequently identified on IncFIB plasmids in MDR *E. coli* isolated from dairy calf feces (5). These plasmids frequently co-encode siderophores and AMR genes (21, 22), providing the biological context for the central hypothesis of this study: the iron-limited preweaned dairy calf gut provides a strong selective force for *E. coli* strains carrying siderophores and, coincidentally, the co-located AMR genes. In *Salmonella* Typhimurium, expression of accessory siderophores, including aerobactin (IucC) and the SitABC transporter, increases in low-iron media, providing a competitive advantage through enhanced iron uptake (19).

To control for the effects of 2,2′-dipyridyl (DIP)-induced iron limitation, all key comparisons of calorimetric output were made between isogenic IncFIB-positive and IncFIB-negative pairs. Based on the observation that plasmid-containing strains were more metabolically active than plasmid-free strains when grown in iron-depleted media, it appears that IncFIB plasmid carriage is critical for *E. coli* growth when iron is scarce. Under iron-replete conditions, plasmid carriage imposed a minor, statistically insignificant, fitness cost; plasmid-bearing strains exhibited slightly lower metabolic activity than their plasmid-free isogenic counterparts (e.g., <15% lower peak heat flow, *P* > 0.05). This suggests a measurable, albeit modest, additional plasmid-carriage burden under nutrient-rich, non-stress conditions. However, when strains were grown under iron limitation, plasmid-bearing strains produced up to 3.5-fold more cumulative heat, reached maximum heat production rates 1.8-fold faster, and maintained metabolic activity 6–10 h longer than plasmid-negative isogenic counterparts (all *P* < 0.01). This pronounced growth advantage effectively offset the associated maintenance cost, confirming that iron restriction provides a strong selective pressure for plasmid-positive cells.

Attribution of the observed physiological benefits to IncFIB plasmid carriage was supported by the transcriptional activity of the plasmid-borne iron acquisition genes. The expression of *sitA, iutA*, and *iucA* was upregulated in both wild-type strains and their transconjugants in iron-depleted growth conditions. While this phenomenon has been previously observed in other non-cecal environments (21, 23), this study represents the first demonstration in bovine cecal media, a natural ecosystem for these organisms. Transcript levels for *iucA* were 11.0-fold to 38.4-fold higher, *iutA* 6.3-fold to 22.7-fold higher, and *sitA* 1.6-fold to 5.3-fold higher across IncFIB+ strains. The presence of these genes on plasmids and chromosomes of other pathogenic and opportunistic Enterobacteriaceae (such as *Salmonella enterica, Klebsiella pneumoniae*, and *Raoultella planticola*) shed by various animals, including humans, and found in many environments underscores their critical role in iron scavenging across diverse ecological niches (24). Thus, these iron-scavenging systems are likely under selection in numerous animal and non-animal environments where iron is limited.

These findings provide a counterpoint to the traditional view that antimicrobial pressure is the sole or primary selective force responsible for AMR persistence (25, 26). Rather, they support the concept of co-selection, wherein AMR genes and iron-acquisition systems are co-encoded on the same plasmid to enable mutual persistence even in the absence of antibiotics. The naturally low iron content of the calf gut favors strains with enhanced iron transport capacity, which in turn inadvertently co-selects strains that carry AMR genes on the same plasmid, thereby promoting the persistence of co-located AMR genes. This effect could be synergistically enhanced by concurrent antimicrobial therapy that directly selects for plasmid-borne resistance genes. Such therapy would provide an additional, direct selective pressure for bacteria harboring IncFIB plasmids encoding AMR genes, compounding the indirect selection already imposed by iron limitation. This observation is consistent with previous studies demonstrating that siderophore-producing bacteria can overcome the intrinsic metabolic fitness cost of plasmid maintenance and maintain competitive advantages and ecological persistence in nutrient-limited environments (27–29). This suggests that in the absence of antibiotic pressure, iron availability acts as a key environmental factor driving the prevalence and persistence of these MDR plasmids in the calf gut.

In addition to the effect of plasmid carriage, there were strain-specific nuances in the response of metabolic activity (as measured by heat flow) to iron limitation. For instance, among the IncFIB-positive wild-type strains under iron depletion, the primary metabolic peaks for two strains were lower than their respective peaks under iron-replete conditions, and there were no secondary peaks of metabolic activity. In contrast, a large secondary metabolic peak was uniquely observed in growing cultures of a third strain. Although there were variations, the difference in metabolic activity for growth in iron-replete vs. iron-depleted conditions was lower in plasmid-bearing strains than in plasmid-free strains. Based on these results, it appears that the IncFIB plasmid generally mitigates iron-stress-induced metabolic depression, with the degree of protection modulated by the host genetic background (21, 22, 30). The metabolism of plasmid-free strains was uniformly suppressed under iron deprivation, whereas plasmid carriers maintained a broader repertoire of metabolic responses, supporting higher average growth-associated heat output.

Results of this study have implications for combating AMR in low-iron environments such as the gut of preweaned dairy calves. Supplementation of colostrum or milk fed to young calves may influence the colonization of siderophore-encoding, antimicrobial-resistant bacteria in the gut. An increase in available ferrous iron in the calf gut may eliminate or reduce the selective advantage conferred by the MDR/siderophore-encoding plasmid in *E. coli* strains and reduce the competition of these plasmid-bearing strains with antimicrobial-susceptible/non-siderophore-encoding *E. coli.* However, formal demonstration of co-selection in this system will require population-level mixed-culture competition experiments. In parallel, refining the iron-limitation model necessitates further validation using iron-rescue assays and alternative chelators. Additionally, comprehensive multi-omic analyses in this system will be needed to fully elucidate plasmid-mediated effects. Finally, the nutritional mitigation strategies proposed here will need to be validated in animal studies that manipulate cecal iron availability and assess the resulting effects on AMR persistence.

## MATERIALS AND METHODS

### Bacterial strains and PCR screening

Eight *E. coli* strains isolated from calf feces were selected as model bacteria for this study: four MDR strains (CC9050, CC11192, CC11237, and CC11251), and four antimicrobial-susceptible strains (CC9710, CC9801, CC11246, and CC11257). All strains were previously characterized for resistance to antimicrobials by automated broth microdilution, and their genomes (including plasmid content) were characterized via whole-genome sequencing (Table 3) (5). Using complete genome assemblies for two representative MDR strains (CC11237 and CC11251), we confirmed that their IncFIB plasmids co-localize the *iucABCD-iutA* and *sitABCD* siderophore operons with multiple antimicrobial resistance loci (Fig. S3 and Table S4). In this study, the presence of iron-acquisition associated genes, *sitA, iucA,* and *iutA*, and the IncFIB replicon was confirmed by interrogating the genomes with VirulenceFinder and PlasmidFinder and subsequently verifying the findings via PCR (31, 32). Primer sequences targeting *sitA, iucA,* and *iutA* and the IncFIB replicon were designed in Primer3Plus (33, 34) (Table 4).

**TABLE 3:** Plasmids and resistance phenotype of bacterial strains (5)^*a*^

Category	Strains	Species	Plasmids (*in silico*)	Resistance phenotype
Multidrug resistance and iron-intake gene-carrying	CC9050	*E. coli*	IncFIA, IncFIB(AP001918), IncI1_Alpha, IncI2_Delta	AMC, AMP, FOX, TIO, CRO, STR, FIS, TET
	CC11192	*E. coli*	Col(MG828), Col(MP18), Col156, Col440I, ColRNAI, IncFIA, IncFIB(AP001918)	AMC, AMP, FOX, CRO, STR, FIS, TET
	CC11237	*E. coli*	IncFIA, IncFIB(AP001918)	AMC, AMP, FOX, CRO, STR, FIS, TET
	CC11251	*E. coli*	Col156, ColRNAI, IncB/O/K/Z, IncFIA, IncFIB(AP001918)	NAL, STR, FIS, TET, SXT
Susceptible and siderophore gene-absence	CC9710	*E. coli*	No hit found	
	CC9801	*E. coli*	No hit found	
	CC11246	*E. coli*	No hit found	
	CC11257	*E. coli*	ColRNAI, IncY	
Intermediate recipient	CC16637	*K. michiganensis*	No hit found	AMP

^*a*^ AMC, amoxicillin-clavulanic acid; AMP, ampicillin; FOX, cefoxitin; TIO, ceftiofur; CRO, ceftriaxone; NAL, nalidixic acid; STR, streptomycin; FIS, sulfisoxazole; TET, tetracycline; SXT, trimethoprim-sulfamethoxazole.

**TABLE 4:** PCR primers used for detection and gene expression assays

Primer name	Target gene/region	Sequence (5′→3′)	Start	Length	Tm (°C)	Product (bp)
Conventional PCR for target detection						
sitA-F-467	*sitA*	ACCTACCAACGTAATGCCGA	467	20	59.1	217
sitA-R-683	*sitA*	CAACCTTACGTACCTGCTGC	683	20	58.9	217
iutA-F-437	*iutA*	GCCTGATCAACATCGTGACC	437	20	59.0	185
iutA-R-621	*iutA*	AAACCAGCCGCCAAATTTCT	621	20	59.0	185
IncFIB-F-83	IncFIB	GATAAGTCGTCCGGTGAGCT	83	20	59.3	167
IncFIB-R-249	IncFIB	AGACGTGTCAGTTCTTCCGT	249	20	59.0	167
16S-F-677	16S rRNA	TCCAGGTGTAGCGGTGAAAT	677	20	59.0	235
16S-R-912	16S rRNA	TGAGTTTTAACCTTGCGGCC	912	20	59.0	235
qPCR for relative gene expression using the 2-ΔΔCt method						
gyrA-F-1304	*gyrA*	AATGGCTGGAGCCAGAGTTC	1,304	20	60.0	81
gyrA-R-1384	*gyrA*	GCAGATCCAGAATCGCCTGA	1,384	20	59.9	81
sitA-F-125	*sitA*	AAAAACGTGGCTGGAGATGC	125	20	59.4	125
sitA-R-249	*sitA*	TTGGCGAGAATCAGTTGTGC	249	20	59.1	125
iutA-F-768	*iutA*	CGACGACGATTACGGGCTTA	768	20	60.0	124
iutA-R-891	*iutA*	GCTGATCAAATGCCGCTCAG	891	20	60.0	124
iucA-F-241	*iucA*	ACCACTCTCTCCCGGCTTAT	241	20	60.0	162
iucA-R-402	*iucA*	CAACGCTTTTTCACGCAGGA	402	20	60.0	162

### Plasmid curing

An ethidium-bromide (EtBr) plasmid-curing method (35, 36) was used to generate mutant derivatives that did not carry the IncFIB plasmid. Each MDR strain was inoculated into Lysogeny broth (BD, Franklin Lakes, NJ, USA) supplemented with EtBr (50, 75, or 125 µg/mL) and incubated at 37 °C for 24 h. Aliquots (200 µL) of each culture were then spread onto LB agar (BD) and incubated for a further 24 h at 37°C to isolate single colonies. One hundred colonies were randomly selected from each plate and replica-plated onto Mueller–Hinton (MH) agar (BD) containing ampicillin (32 µg/mL), streptomycin (16 µg/mL), or tetracycline (32 µg/mL), and onto drug-free MH agar, followed by incubation at 37°C for 24 h. Colonies that had lost resistance to at least one antimicrobial were designated as putative plasmid-cured isolates. The EtBr exposure was extended to 48 h if no candidates were obtained, and the screening procedure was repeated. Loss of the IncFIB replicon and the iron-acquisition genes (*iucA, iutA,* and *sitA*) was confirmed in all putative isolates by PCR using the primer sets listed in Table 4. Isolates negative for both the plasmid marker and these siderophore genes were designated the final plasmid-cured derivatives. The results of plasmid curing experiments are described in Table S1.

### Two-step conjugation via *K. michiganensis*

To evaluate the functional contribution of the IncFIB plasmid, we transferred it from four MDR donor strains to plasmid-negative recipients, either antimicrobial-susceptible strains or plasmid-cured derivatives. A modified two-step conjugation protocol was employed due to the lack of selectable markers in the susceptible recipients (23, 37–39). This involved using *K. michiganensis* CC16637 (GenBank accession JAVXUQ000000000.1) as an intermediate host. Mid-log phase cultures of each MDR *E. coli* donor and *K. michiganensis* were mixed at a 1:1 volumetric ratio and co-incubated for 4–6 h at 37°C with shaking (200 rpm). The mixtures were then streaked onto Simmons citrate agar (Neogen, Lansing, MI, USA) containing tetracycline (16 µg/mL) to select tetracycline-resistant *K. michiganensis* transconjugants. Putative *Klebsiella* colonies were verified on CHROMagar Orientation (CHROMagar, Paris, France) and Simmons citrate agar. Plasmid acquisition was confirmed by PCR targeting the IncFIB replicon and the iron-acquisition genes *iucA*, *iutA,* and *sitA*. Verified *K. michiganensis* transconjugants were subsequently mated with plasmid-negative *E. coli* recipients. Post-mating cultures were plated on agar each supplemented with a single antibiotic: ampicillin (16 µg/mL), streptomycin (16–32 µg/mL), tetracycline (8–16 µg/mL), or chloramphenicol (0.5–1 µg/mL) and incubated at 37°C and 44°C for 24 h (extended to 48 h if no colonies appeared) (40). In instances where colony overlap occurred, subculturing was conducted to isolate single colonies onto the antimicrobial agars described above. Surviving colonies were presumptively identified as *E. coli* based on their characteristic colony morphology on CHROMagar Orientation. Subsequent PCR analysis was used to confirm both the species identification and the successful acquisition of the IncFIB plasmid. These confirmed IncFIB-positive derivatives were preserved for downstream analyses. The results of conjugation experiments are described in Table S2.

### Cecal medium preparation

To mimic the bovine cecal environment, fresh cecal contents were collected from six culled dairy cows and pooled. Briefly, 30 g of cecal material from each cow was placed in a filter bag with 270 g of PBS (pH 7.4) and homogenized. The homogenate was centrifuged at 9,000 × *g* for 90 min at 22°C, and the supernatant was sequentially passed through sterile 0.45-µm and 0.22-µm cellulose-acetate filters. Equal volumes of the filtered preparations were combined to generate a 10% (wt/vol) “mixed six-cow cecal medium.” Due to the inability to procure preweaned calf cecal media, we used pooled adult-bovine cecal filtrate as a reproducible bovine cecal matrix for comparative testing. Iron restriction in this medium was imposed by adding 2,2′-dipyridyl (DIP; final concentration 400 µM). We refer to these conditions throughout as DIP-mediated iron-restricted bovine cecal medium, which is intended to mimic key aspects of the low-iron environment of the preweaned calf gut. Aliquots from this single-pooled batch were used for all experiments, so that all assays were conducted within the same standardized cecal matrix. All media were stored at 4°C until use.

### Siderophore gene expression analysis

Expression of plasmid-encoded iron-acquisition genes (*sitA*, *iutA*, and *iucA*) was analyzed by RT-qPCR. The four wild-type MDR and the six transconjugant strains carrying an IncFIB plasmid were cultured in 10% cecal medium under normal and iron-depleted conditions. Cultures were harvested at mid-logarithmic phase (OD600 ≈ 0.5–0.7). Following cell harvest, the samples were treated with RNAprotect Bacteria Reagent (QIAGEN, Hilden, Germany) to stabilize the RNA, followed by total RNA extraction using the RNeasy Protect Bacteria Mini Kit (QIAGEN). Complementary DNA (cDNA) was synthesized using the Maxima First Strand cDNA Synthesis Kit for RT-qPCR with dsDNase (Thermo Scientific, Waltham, MA, USA) in a 20 µL reaction. The *gyrA* gene was used as the internal reference control. Primers specific to targeted genes were designed using Primer3Plus (34), and qRT-PCR was performed using PowerUp SYBR Green Master Mix (Thermo Scientific) on a Stratagene Mx3005P real-time PCR system (Agilent Technologies, Santa Clara, CA, USA). Each reaction (20 µL) contained 10 µL of SYBR Green Master Mix, 0.5 µL of each primer (10 µM), 2 µL of cDNA, and 7 µL of RNase-free water. Thermal cycling conditions included denaturation at 50°C for 2 min, 95°C for 2 min, followed by 40 cycles of 95°C for 15 s and 60°C for 60 s, with melting curve analysis from 65°C to 95°C in 0.5°C increments. Relative gene expression was calculated using the 2^-^*^ΔΔ^*^Ct^ method, normalized to *gyrA*. No-template controls and no-reverse-transcription controls confirmed the absence of contamination. Triplicates were analyzed per strain under each condition (41). Statistical significance of expression differences (iron-depleted vs. normal) was assessed with two-tailed Welch’s *t*-tests on replicate-level *Δ*C_t_ values; *P* < 0.05 was considered significant.

### Validation of isothermal microcalorimetry (IMC) against standard growth metrics

To ensure that IMC is a reliable method for monitoring bacterial growth, a foundational experiment was conducted to compare the heat production data directly with traditional methods. *E. coli* ATCC 25922 was revived from a frozen stock on MacConkey II agar (BD) and then grown in M9 minimal broth (5× minimal salts [BD] and supplemented with 2 mM MgSO_4_, 0.1 mM CaCl_2_, and 0.4% [wt/vol] D-(+)-glucose). Following a secondary streak on MacConkey II agar to ensure purity, three colonies were used to inoculate a pre-culture. This overnight culture was then diluted 1:100 into a single 500 mL homogenized main culture, which served as the sole source for all parallel experiments.

**Fig 5:**
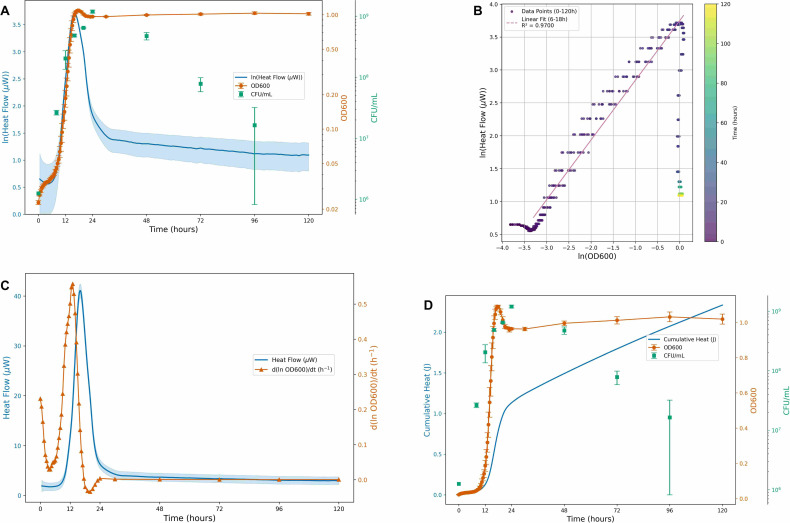
Isothermal microcalorimetry accurately reflects the multiphasic growth dynamics of *E. coli*. Comparative analysis of *E. coli* ATCC 25922 growth monitored by isothermal microcalorimetry (IMC), spectrophotometry (OD600), and viable cell counting (CFU). All lines and error bars represent the mean ± standard deviation of *N* replicates (*N* = 23 for IMC, *N* = 12 for OD600, *N* = 3 for CFU). (**A**) Temporal profiles of ln(Heat Flow), log-transformed OD600, and CFU, showing high visual similarity in their overall growth patterns. (**B**) Log–log scatter plot of ln(ϕ) versus ln(OD600) with a regression line. The strong linear relationship during the balanced (mid-exponential) growth windows (6–18 h, R² = 0.9700) supports the direct relationship between metabolic heat production and biomass. (**C**) Comparison of the real-time heat flow curve and the specific growth rate (d(lnOD600)/dt). The peak of the specific growth rate (13.0 h) precedes the peak of total metabolic activity (16.03 h), accurately capturing the transition from exponential growth. (**D**) Comparison of total metabolic output (cumulative heat) with total biomass (OD600) and viability (CFU).

Growth was monitored simultaneously using three methods: IMC, spectrophotometry (OD600), and viable cell count (CFU/mL). For IMC, 200 µL culture aliquots were dispensed into 23 experimental and nine blank vials for continuous heat flow (µW) analysis in a CalScreener (Symcel, Stockholm, Sweden). For spectrophotometry, optical density at 600 nm (OD600) was measured in 12 replicate tubes (10 mL culture) using a Genesys 30 spectrophotometer (Thermo Scientific) at defined intervals up to 120 h. For viable cell counts, a separate set of 24 test tubes was prepared for destructive sampling at selected time points (8, 12, 16, 20, 24, 48, 72, and 96 h) to determine cell count (CFU/mL) using an easySpiral Pro plater (Interscience, Saint Nom, France). Data from OD600, CFU/mL, and heat flow (µW) were subsequently compared to analyze the relationship between cell density, cell count, and metabolic activity (see Results, Fig. 5).

### Data analysis and theoretical framework

The relationship between biomass (*X*, from OD600), viability (CFU), and metabolic activity (heat flow, ϕ) was analyzed using the fundamental principle that total heat flow is proportional to the product of biomass and the specific heat production rate per cell (*q*) (42, 43)


(1) ϕ≈q×X

This relationship can be linearized by a natural logarithm, making it additive:


(2) ln⁡ϕ≈ln⁡q+ln⁡X

This logarithmic framework predicts that during balanced (mid-exponential) growth, when cellular composition and the per-cell heat production rate *q* are approximately constant, ln(ϕ) should be linearly correlated with ln(OD600). This principle was used to validate the IMC data against standard growth metrics.

The overall profiles of ln(Heat Flow) and log-transformed OD600 were visually similar over time, and their respective peak times were in close agreement (16.03 h and 17.5 h, respectively) (Fig. 5A). To quantitatively test proportionality, we fitted a log–log linear regression of ln(ϕ) on ln(OD600) within the balanced-growth window (6–18 h). As predicted by the theoretical framework, the fit showed a strong linear relationship (R² = 0.9700; Fig. 5B). Furthermore, IMC captured the detailed dynamics of the growth phase transition. The point of most rapid deceleration in the specific growth rate, calculated from the derivative of ln(OD600), occurred at 15 h and 15.5 h, immediately preceding the time of peak total metabolic activity measured by IMC (16.03 h). This indicates that the heat flow peak is tightly coupled with the physiological moment the population transitions from exponential to stationary phase (Fig. 5C).

Finally, when comparing total output, the sigmoidal profile of the cumulative heat curve mirrored the growth patterns shown by both OD600 and CFU counts. Notably, in the late stationary phase, IMC continued to detect residual metabolic heat production even after CFU began to decline, a phenomenon not captured by the OD600 plateau (Fig. 5D). Collectively, these results confirm that IMC provides a reliable, real-time measure of bacterial growth dynamics, showing strong correlations with standard metrics of biomass and viability.

### IMC for iron-fitness assays

Real-time metabolic activity of the eight *E. coli* strains and their derivatives was monitored with a multichannel isothermal calorimeter (CalScreener IMC; Symcel). To prepare the inoculum, test strains were first grown overnight in 10% cecal medium to ensure they reached the stationary phase. These cultures were then subcultured (1:100) into fresh 10% cecal medium and incubated for approximately 6 h to obtain cells in a consistent mid-logarithmic growth phase. This procedure to standardize the physiological state of the cells was followed by a 10⁻⁷ serial dilution in either untreated 10% cecal medium (normal) or 10% cecal medium supplemented with 400 µM DIP to prepare the final inoculum. This dilution was chosen to provide a stable thermal baseline and minimize physiological carryover. Aliquots (200 µL) of the final diluted cultures were loaded in triplicate into CalScreener inserts (Symcel). Heat-flow signals were recorded continuously and exported at a 1-s interval. Potential minor variations in the starting cell density among strains were accounted for during data analysis by aligning all kinetic curves relative to their respective time to activation (TTA) (see “Processing and multivariate analysis of IMC data” section).

### Processing and multivariate analysis of IMC data

Raw heat-flow data from the iron-fitness assays were processed using a custom workflow in R (v4.3.2) (Fig. S1). After excluding the initial 30-min instrument equilibration phase, data were smoothed using a spline function (spar = 0.3), and a dynamic baseline correction was applied based on a stable 600-s pre-peak segment. Cumulative heat (Q(t)) was calculated using the trapezoidal integration method. From the corrected heat flow (ϕ(t)) and cumulative heat (Q(t)) curves, a comprehensive set of twelve growth kinetic metrics was derived. These metrics were divided into those describing the early-growth phase (e.g., TTA, maximum rate of increase in heat flow [μ_max_], and doubling time) and the late-growth phase (e.g., decay rate and heat-flow width indicators). The precise definition and calculation method for each metric are provided in Table S3.

To visualize overarching patterns in the data and specifically test our hypothesis regarding the initial response to iron stress, a multivariate analysis was performed. For this analysis, we selected the seven early-phase metrics described above, as they directly pertain to the initial growth dynamics. This subset of metrics was first Z-standardized (mean = 0, variance = 1) and then subjected to principal-component analysis (PCA). The same normalized matrix served as input for a permutational multivariate analysis of variance (PERMANOVA) using Bray–Curtis distances and 9,999 permutations to test the main effects of plasmid status, medium, and their interaction.

To further probe functional heterogeneity among IncFIB plasmids, a second PCA was carried out on the iron-stress response matrix (*Δ* = Depleted – Normal). The five variables that contributed most strongly to the first PCA were combined with the log₂ fold-change of *iucA* obtained by RT-qPCR, Z-scaled, and analyzed separately for iron-replete and iron-depleted media.

### Conclusion

The integrated approach, combining high-resolution isothermal microcalorimetry and gene expression analysis, supports the hypothesis that iron limitation in the preweaned calf gut indirectly selects for MDR *E. coli* strains carrying plasmid-encoded siderophore systems. Under iron-scarce conditions, these strains gain a distinct fitness advantage over IncFIB plasmid-negative strains. This advantage simultaneously promotes the colonization of co-located AMR genes, even in the absence of direct antimicrobial pressure.

## Data Availability

The genome sequence of strain *Klebsiella michiganensis* CC16637 has been deposited in the GenBank database with the accession number JAVXUQ000000000.1. Genomic data for *Escherichia coli* strains CC11237 and CC11251 were deposited under BioSample IDs SAMN37429623 and SAMN37429637, respectively.

## SUPPLEMENTAL MATERIAL

Supplemental material aem.01952-25-s0001.docx Fig. S1 to S3, Tables S1 to S4, and Method S1.

ASM does not own the copyrights to Supplemental Material that may be linked to, or accessed through, an article. The authors have granted ASM a non-exclusive, world-wide license to publish the Supplemental Material files. Please contact the corresponding author directly for reuse.
