# Supplementary material for: A nutritional link to antimicrobial resistance: iron scarcity promotes plasmid co-selection in Escherichia coli
Source: Appl Environ Microbiol. 2026 May 6;92(6):e01952-25. doi: 10.1128/aem.01952-25 (PMC13274395; doi:10.1128/aem.01952-25)
Supplement: Supplemental material — Fig. S1 to S3, Tables S1 to S4, and Method S1. [file aem.01952-25-s0001.docx]

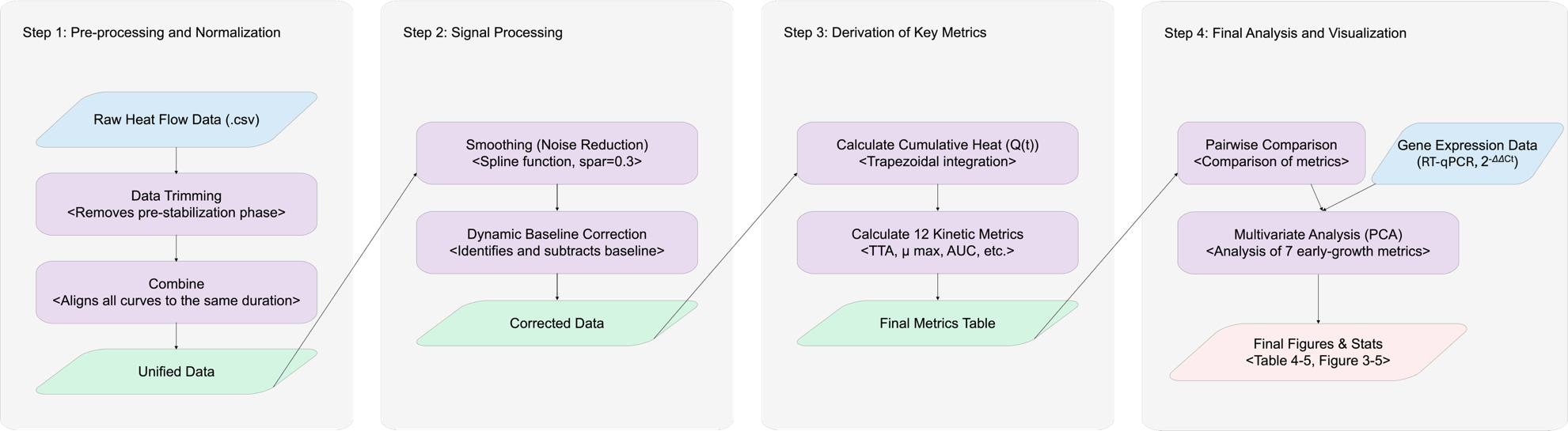


# Supplementary Figure S1. Custom R workflow for the processing and analysis of isothermal microcalorimetry (IMC) data.

This flowchart summarizes the four-step computational pipeline used to derive final growth kinetic metrics and statistical analysis results from raw heat flow data (.csv). All analyses were performed in the R environment (v4.3.2), leveraging the data.table package for efficient data handling and the future.apply and parallel packages for parallel processing.

**Step 1: Pre-processing and normalization.** For each raw time-series sample, the late-stage stabilization phase following the main growth peak is identified to trim the data. Subsequently, the length of all samples is unified to that of the shortest curve in the dataset, normalizing the time axis for all curves.

**Step 2: Signal processing.** First, minor noise in each curve is removed using the stats::smooth.spline function (smoothing parameter spar=0.3). Next, a dynamic baseline is defined by identifying the most stable 600-second segment within the pre-peak phase, based on criteria of minimal variance and a slope of ≤ 1x10⁻⁶ µW/s. The mean value of this baseline segment is then subtracted from the entire curve for correction.

**Step 3: Derivation of key metrics.** The corrected heat flow curve (ϕ(t)) is integrated over time using the trapezoidal integration method to calculate the cumulative heat curve (Q(t)). From these two curves, all twelve growth kinetic metrics described in the main text are extracted, including the **Time-to-Activation (TTA)**, which is derived from the tangent at the point of maximum acceleration on the Q(t) curve.

**Step 4: Final analysis and visualization.** The full set of 12 derived metrics is used for direct statistical pairwise comparisons between isogenic strain pairs. Additionally, for hypothesis testing, a subset of seven early-growth phase metrics is Z-standardized and subjected to multivariate analysis, including principal-component analysis (PCA, stats::prcomp function) and permutational multivariate analysis of variance (PERMANOVA, vegan::adonis2 function). To further explore heterogeneity among plasmid-positive strains, a subsequent PCA is performed by integrating the top five growth metrics with *iucA* gene expression data from RT-qPCR. This entire process generates the key tables (Tables 4-5) and figures (Figures 3-5) of the manuscript.

# Supplementary Table S1. Result of plasmid curing

|  | CC9050 | CC11192 | CC11237 | CC11251 |
| --- | --- | --- | --- | --- |
| Curing rate | 4% | 1% | 25% | 2% |

When *E. coli* isolates were treated with ethidium bromide to induce plasmid loss, the plasmid curing efficiency for AMR and siderophore-encoding *E. coli* strains CC9050, CC11192, CC11237, and CC11251 ranged from a low of 1% to a high of 25%. Although the plasmid curing efficiencies were low, the plasmid was successfully removed from each strain. PCR screening of the cured derivatives confirmed successful plasmid loss by yielding no amplicons for the IncFIB replicon marker or the siderophore genes *sitA* and *iutA*. For the purpose of this study, the plasmid-cured derivatives of CC9050, CC11192, CC11237, and CC11251 are designated as CC9050Cured, CC11192Cured, CC11237Cured, and CC11251Cured, respectively.

# Supplementary Table S2. Result of two-step conjugation via *Klebsiella michiganensis*

|  | Cured Strains | | | | Susceptible Strains | | | |
| --- | --- | --- | --- | --- | --- | --- | --- | --- |
| Recipients | CC9050Cured | CC11192Cured | CC11237Cured | CC11251Cured | CC9710 | CC9801 | CC11246 | CC11257 |
| Donors |  |  |  |  |  |  |  |  |
| *Klebsiella*::pCC9050 |  | O |  |  |  |  |  |  |
| *Klebsiella*::pCC11192 | O |  |  |  | O | O | O |  |
| *Klebsiella*::pCC11237 |  |  |  |  |  |  |  |  |
| *Klebsiella*::pCC11251 |  | O |  |  |  |  |  |  |

O = transconjugant recovered; blank = transconjugants could not be recovered.

While the initial plasmid transfer from the four MDR *E. coli* donors to the *K. michiganensis* intermediate was successful in all cases, the subsequent transfer to the final *E. coli* recipients was not always successful. A total of six *E. coli* transconjugants were successfully generated using the two-step conjugation method (Table S2).

The six successful transconjugants were comprised of two distinct recipient groups. The first group consisted of three transconjugants generated using naturally susceptible strains as recipients: CC9710::pCC11192, CC9801::pCC11192, and CC11246::pCC11192, which all received a plasmid from donor CC11192. The second group consisted of three transconjugants generated using plasmid-cured derivatives as recipients: CC9050Cured::pCC11192, CC11192Cured::pCC9050, and CC11192Cured::pCC11251.

# Supplementary Table S3. Definitions, calculation methods, and key references for kinetic metrics derived from isothermal microcalorimetry (IMC) data for microbial growth analysis.

| Metric (Abbreviation) | Definition | Rationale / Interpretation | Citation |
| --- | --- | --- | --- |
| Early-Growth Phase Metrics |  |  |  |
| Time to activate (TTA) | The intercept on the time-axis of a tangent drawn to the point of maximum acceleration (inflection point) on the cumulative heat curve, Q(t). | Represents the onset of detectable metabolic activity and quantifies the lag phase duration. | (1, 2) |
| Lag phase | The time from the start of the culture (t=0) to TTA. | The period required for microorganisms to adapt to the new environment and synthesize enzymes and metabolites necessary for growth. TTA serves as a biophysical marker for the end of the lag phase. | (2) |
| ****Relative time of peak heat flow (TP)**** | The time elapsed from TTA to the point where the heat flow curve, P(t), reaches its maximum value. | The point of peak metabolic activity, often coinciding with the transition from the exponential to the stationary phase. It can reflect the onset of nutrient depletion or the accumulation of metabolic byproducts. |  |
| ****μ_max_**** | The maximum value of the first derivative of the heat flow curve. (maximum rate of increase in heat flow) | Shows a high positive correlation with the maximum specific growth rate μ during the exponential phase. It is a kinetic landmark representing the highest rate of increase in metabolic activity. | (1) |
| ****Tμ_max_**** | The time, relative to TTA, at which the **μ_max_** occurs. | Marks the midpoint of the exponential growth phase where metabolic acceleration is fastest. It is a physiologically significant point indicating the transition from unrestricted to resource-limited growth. | (1, 2) |
| Doubling time | The time required for the cumulative heat, Q(t), to double during the exponential phase immediately following TTA. | Since cumulative heat (Q) is a proxy for total biomass (X), this time reflects the classic doubling time required for the cell population to double. | (1, 2) |
| Area under the curve of pre-peak area | The area under the heat flow curve, from TTA to TP. | The total amount of energy released during the exponential growth phase. |  |
| Late-Phase Metrics |  |  |  |
| Decay rate | The slope of the curve after TP as the heat flow stabilizes. | Represents the rate of decrease in metabolic activity during the stationary or death phase. |  |
| Peak Width (FW90 / FW95 / FWHM) | The time duration for which the heat flow signal remains above 90%, 95%, or 50% (Full Width at Half Maximum) of its maximum peak height, respectively. | Measures the duration for which the highest level of metabolic activity is sustained. | (1, 2) |
| AUC of Post-Peak | The area under the heat flow curve, from TTA up to a specified time X (e.g., 24, 36, 48 h). | The total energy output over a specific culture duration. It can be used to assess long-term metabolic viability, secondary metabolite production, or survival capacity under stress conditions. |  |


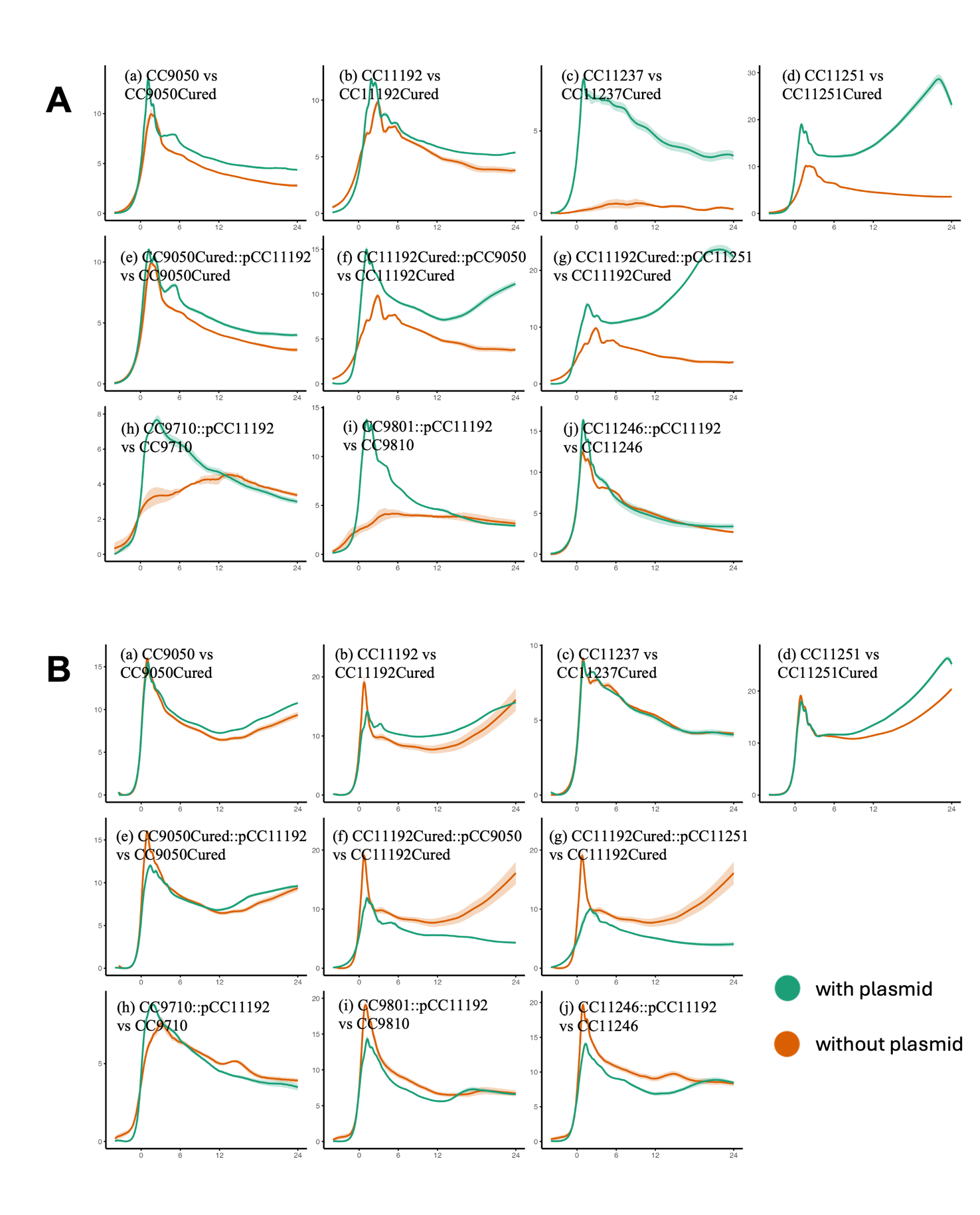


# Supplementary Figure S2. ****Real-time heat-flow profiles of ten**** Escherichia coli ****strain pairs grown in iron-depleted and normal medium.****

**A**, in iron-depleted medium; **B**, in normal medium

**Group 1: WT MDR vs IncFIB cured MDR**

(a) CC9050 vs CC9050Cured; (b) CC11192 vs CC11192Cured; (c) CC11237 vs CC11237Cured; (d) CC11251 vs CC11251Cured;

**Group 2: IncFIB reintroduction vs IncFIB cured MDR**

(e) CC9050Cured::pCC11192 vs CC9050Cured; (f) CC11192Cured::pCC9050 vs CC11192Cured; (g) CC11192Cured::pCC11251 vs CC11192Cured;

**Group 3: Transconjugant vs WT susceptible**

(h) CC9710::pCC11192 vs CC9710; (i) CC9801::pCC11192 vs CC9810; (j) CC11246::pCC11192 vs CC11246

# Supplementary Figure S3. Maps of IncFIB plasmids from MDR *Escherichia coli* strains CC11237 and CC11251.

**(A)**


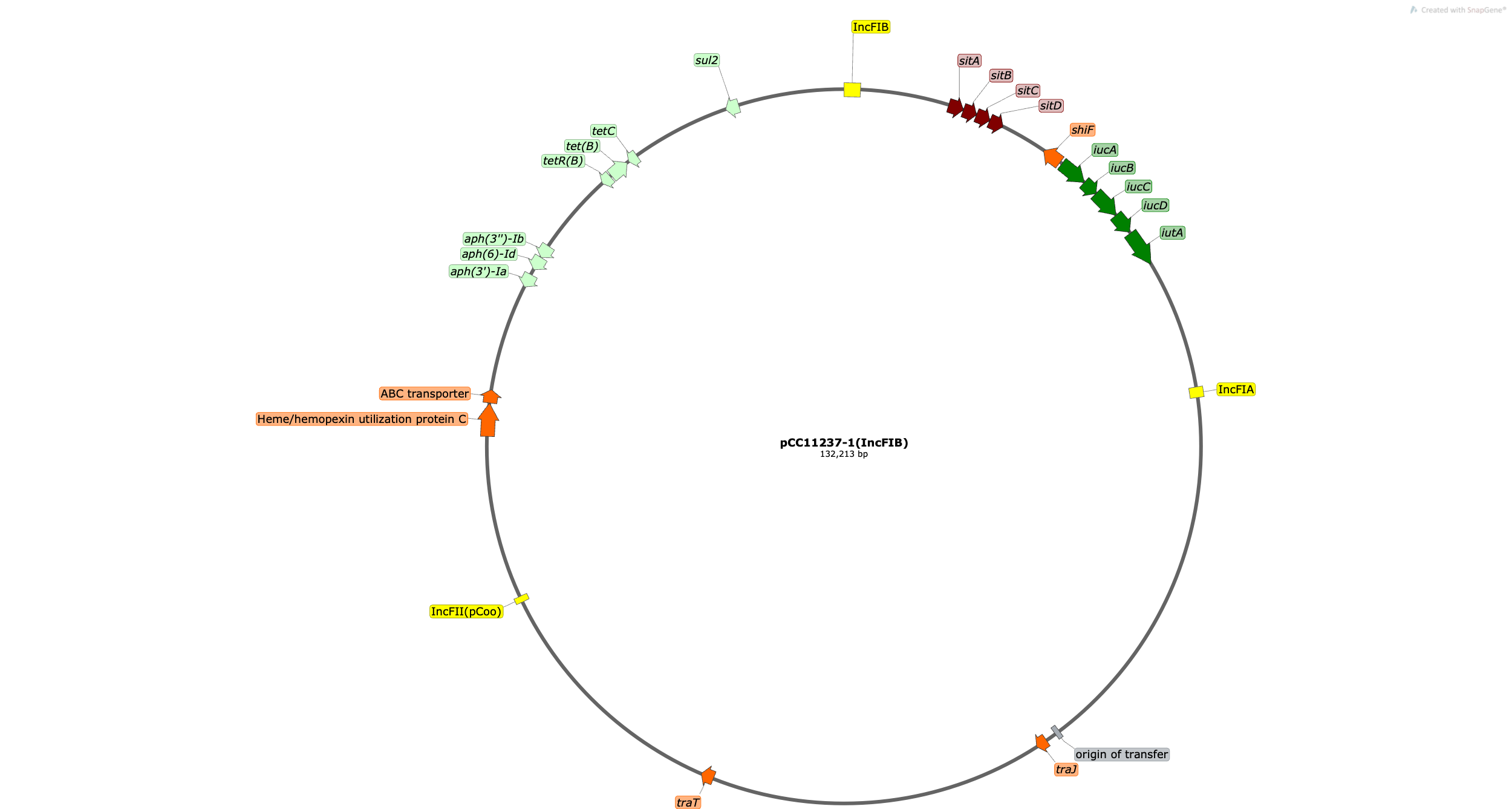


**(B)**

#
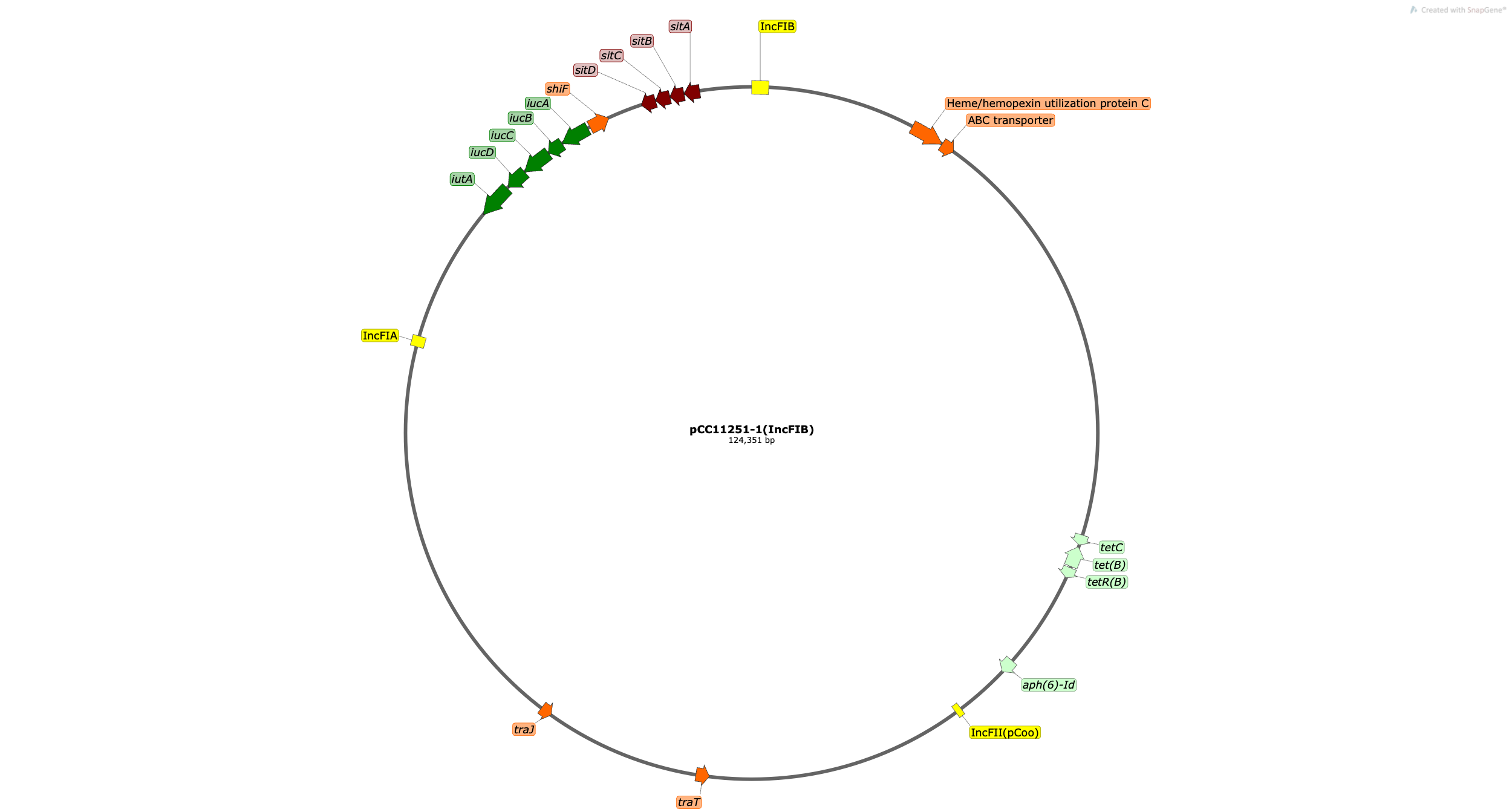


(A) pCC11237-1 and (B) pCC11251-1. The maps highlight the physical co-localization of the siderophore and iron-uptake gene clusters *iucABCD–iutA* (aerobactin system) and *sitABCD* with multiple antimicrobial resistance loci on the same IncFIB plasmid. Replicon markers (IncFIB, IncFIA, and IncFII) and transfer-associated features are annotated where present.

*Siderophore and iron-uptake genes (green and red), antimicrobial resistance genes (light green), replicon markers (yellow), and other accessory genes (orange).

# Supplementary Table S4. Co-occurrence of strict siderophore operons and AMR genes among IncFIB-positive plasmids (NCBI public genomes)

| IncFIB+ only | AMR− | AMR+ |
| --- | --- | --- |
| Siderophore- | 2,760 | 2,732 |
| Siderophore+ | 387 | 1,155 |

Odds ratio (Fisher) ≈ 3.01; 95 percent CI approximately 2.7–3.4; p < 10⁻⁵⁰. AMR, presence of at least one antimicrobial resistance gene; Siderophore+, plasmids carrying both complete aerobactin (*iucABCD*–*iutA*) and sit (*sitABCD*) operons; IncFIB+, plasmids positive for the IncFIB replicon.

75 percent of siderophore-full-set-positive IncFIB plasmids were AMR-positive (1,155/1,542), compared with 50 percent of siderophore-full-set-negative IncFIB plasmids (2,732/5,492), indicating a strong within-IncFIB association between strict siderophore modules and AMR loci.

# Supplementary Method S1. Public plasmid database survey of IncFIB-associated siderophore and antimicrobial resistance modules

We compiled a public reference set of *E. coli* plasmids and used it to quantify co-occurrence between siderophore and AMR modules at the plasmid level. First, we queried the NCBI nucleotide database for *Escherichia coli* plasmid records using the Entrez utilities and restricted the results to entries annotated as complete plasmid sequences, excluding whole genome shotgun contigs, scaffolds, and non plasmid replicons. After filtering, the final dataset comprised 28,697 unique, complete plasmid sequences.

These plasmid sequences were screened with ABRicate (version 1.0.1) using the PlasmidFinder, ResFinder, and *E. coli* virulence factor (ecoli_vf) databases, all downloaded and indexed on 25 November 2025. IncFIB positive plasmids were defined as those with at least one IncFIB replicon hit in PlasmidFinder. AMR positive plasmids (AMR⁺) were defined as those carrying at least one ResFinder antimicrobial resistance determinant. For siderophore loci, we used the ecoli_vf database to detect aerobactin (*iucABCD* and *iutA*) and sit (*sitABCD*) operons. In the strict analysis, “siderophore-full-set” plasmids were defined as those carrying a complete aerobactin operon (*iucA, iucB, iucC, iucD*, and *iutA*) and a complete sit operon (*sitA, sitB, sitC*, and *sitD*). Plasmids missing any of these components were classified as “siderophore fullset” negative.

ABRicate output files (PlasmidFinder, ResFinder, and ecoli_vf) were summarized at the plasmid level to record, for each plasmid, IncFIB replicon status, strict siderophore fullset status, and AMR status. We then restricted the analysis to IncFIB positive plasmids (7,034 sequences) and constructed a 2×2 contingency table with AMR status as rows (AMR- and AMR+) and strict siderophore fullset status (negative and positive) as columns. Estimates and statistical results from this analysis are reported in Supplementary Table S4.

# References

1. Braissant O, Bonkat G, Wirz D, Bachmann A. 2013. Microbial growth and isothermal microcalorimetry: Growth models and their application to microcalorimetric data. Thermochimica Acta 555:64-71.

2. Braissant O, Wirz D, Göpfert B, Daniels AU. 2010. Use of isothermal microcalorimetry to monitor microbial activities. FEMS microbiology letters 303:1-8.

3. ABRIcate, <https://github.com/tseemann/abricate>.
